# Practical Hygiene

**Published:** 1906-09

**Authors:** 


### BOOK REVIEWS.

Practical Hygiene, By C. Gilman Currier, M. D. Visiting Physician to the New York City Hospitals, Fellow of the New York Academy of Medicine, Member of the American Medical Association, etc., etc. Fifth edition revised and enlarged, 8vo, 488 pages, illustrated, cloth $2.00. 1905. Publishers, E. B. Treat & Co, 241 W. 23rd St. N. Y.

The intelligent and scientific utilization of all known means for prolonging life and preventing diseases has become a specialty in itself. Preventive Medicine is not only to be the Medicine of the future but it is fast becoming the Medicine of today.

This volume was prepared in recognition of this fact and in response to a large an dgrowing demand and upon investigation it will be found to embody the results of the most profound research. It is by far the most recent, scientific, agreeably written and practically instructive of all the existing books on the subject.

